# Mie metasurfaces for enhancing photon outcoupling from single embedded quantum emitters

**DOI:** 10.1515/nanoph-2024-0300

**Published:** 2024-10-17

**Authors:** Samuel Prescott, Prasad P. Iyer, Sadhvikas Addamane, Hyunseung Jung, Ting S. Luk, Igal Brener, Oleg Mitrofanov

**Affiliations:** Electronic and Electrical Engineering, 4919 University College London, London, WC1E 7JE, UK; Center for Integrated Nanotechnologies, Sandia National Labs, Albuquerque, NM, USA; Sandia National Laboratories, Albuquerque, NM, USA

**Keywords:** single photon source, metasurface, quantum information, quantum dot, quantum emitter, Mie resonator

## Abstract

Solid-state quantum emitters (QE) can produce single photons required for quantum information processing. However, their emission properties often exhibit poor directivity and polarisation definition resulting in considerable loss of generated photons. Here we propose and numerically evaluate Mie metasurface designs for outcoupling photons from an embedded and randomly-positioned QE. These Mie metasurface designs can provide over one order of magnitude enhancement in photon outcoupling with only several percent of photons being lost. Importantly, the Mie metasurfaces provide the enhancement in photon outcoupling without the need for strict QE position alignment and without affecting the intrinsic QE emission rate (Purcell enhancement). Electric dipole modes are key for achieving the enhancement and they offer a path for selective outcoupling for photons emitted with specific polarisation, including the out-of-plane polarisation. Mie metasurfaces can provide an efficient, polarisation-selective and scalable platform for QEs.

## 1 Introduction

Quantum information applications, including quantum computation and cryptography, require single-photon sources [1], [2], [3] with efficient free-space photon outcoupling for information processing [4]. Among many types of quantum emitters (QE), solid-state based atom-like systems [3], such as semiconductor quantum dots (QDs) [5], [6], [7], [8] and atomic defects in crystals [9], [10], [11], promise the potential for monolithic integration and scalability [12], [13], [14], [15], [16]. However, intrinsic properties of most solid-state quantum sources present two major challenges for free-space photon outcoupling: (1) the spontaneous emission rate is low for practical quantum information applications, and (2) the emission pattern exhibits poor directivity and polarisation definition resulting in only a small fraction of generated photons coupling to free space.

A powerful approach to address these challenges is to engineer the near-field environment of the QE [4]. By placing the emitter within a photonic crystal cavity [17], [18], a micropillar [19], or a ring resonator [20], the spontaneous emission rate can be increased by over one order of magnitude via the Purcell effect [21], whereas coupling an emitter to a plasmonic nano-antenna [22], [23], [24] or a specially-designed metasurface [25], [26], [27], [28], [29], [30] can produce directional single-photon beams with desired polarisation and structure. This approach however often requires high levels of precision nano-scale fabrication [31] and/or multiple lithography rounds limiting their repeatability and scalability. Recently, dielectric Mie metasurfaces were proposed for improving the outcoupling while simultaneously shaping the emission directivity [32], [33], [34]. Due to the periodic nature of Mie metasurfaces, the requirement on precise placement of the QE can be relaxed, whereas the outcoupling can be enhanced by over one order of magnitude for a single emitter [35]. Furthermore, the nature of Mie modes has the potential to enable preferential outcoupling of photons from a QE with specific dipole moment orientation, thus providing a path for tailoring the metasurface design to the intrinsic emission properties of QEs and generating highly directional single-photon beams with desired polarisation and structure. However, the role of various Mie modes in emission enhancement for QEs with a specific dipole moment orientation and the underlying mechanisms have not been investigated.

Here, we quantify the effect of fundamental electric and magnetic dipole Mie modes and their orientation on the photon emission outcoupling efficiency for a single QE embedded in the metasurface numerically, taking into account the emitter polarisation properties, randomness in its position within the Mie resonator as well as the practical factors such as the numerical aperture of collection optics. Using FDTD simulations, we evaluate the effect of two metasurface designs, where a QE is coupled to (1) a combination of the fundamental in-plane electric dipole (ED) and magnetic dipole (MD) Mie modes (the combination known as Huygens’ metasurface [35], [36], [37]); and (2) a combination of two degenerate in-plane and out-of-plane ED modes. The former (Huygens’) metasurface design can facilitate free-space photon outcoupling for QEs with omnidirectional orientation of its dipole moment, such as W-defects in silicon [10], and emitters with the dipole moment orientation in the metasurface plane, for example the low-density epitaxial semiconductor QDs [38]. The second design can further facilitate preferential outcoupling for QEs with out-of-plane dipole moment orientation, such as stacked epitaxial QDs [39], [40] and defects in ZnO [41].

We find that both metasurface designs increase the emission from a single embedded emitter into free space by over one order of magnitude when the emission wavelength aligns spectrally with the metasurface modes. For the Huygens’ metasurface, in particular, the outcoupling efficiency into the air region for an in-plane polarised embedded QE can reach up to 35 % when the ED and MD modes overlap with the emission wavelength, compared to the efficiency of under 1 % for QEs embedded in a dielectric slab, which traps over 90 % of photons. For the second metasurface with the out-of-plane ED mode, the outcoupling efficiency into air can reach up to 36 % for photons with the out-of-plane polarisation. Additionally, we found that the average Purcell enhancement remains approximately unity (with some variation, up to 1.5, due to the QE position within metasurface resonators), indicating that the QE intrinsic emission rate remains weakly affected by the Mie metasurface. Therefore, the emission enhancement enabled by the Mie metasurfaces is due to efficient outcoupling of photons which are otherwise trapped in the substrate.

The semiconductor Mie metasurface platform can significantly improve extraction of single-photon emission without the need for strict QE spatial position alignment, while also enabling selective enhancement for photons emitted with desired in-plane and out-of-plane polarisations. Our results reveal the physics behind the recent experimentally observed emission enhancement for the low-density local droplet etched (LDE) epitaxial GaAs QDs embedded in AlGaAs Mie metasurfaces [33]. For epitaxial QDs, the Mie metasurface can enable monolithic and scalable QD integration while offering a multitude of functionalities, including the control of emission polarisation and directionality, and the control over supported resonance Q-factors [42].

## 2 Metasurface design concept

**Figure 1:**
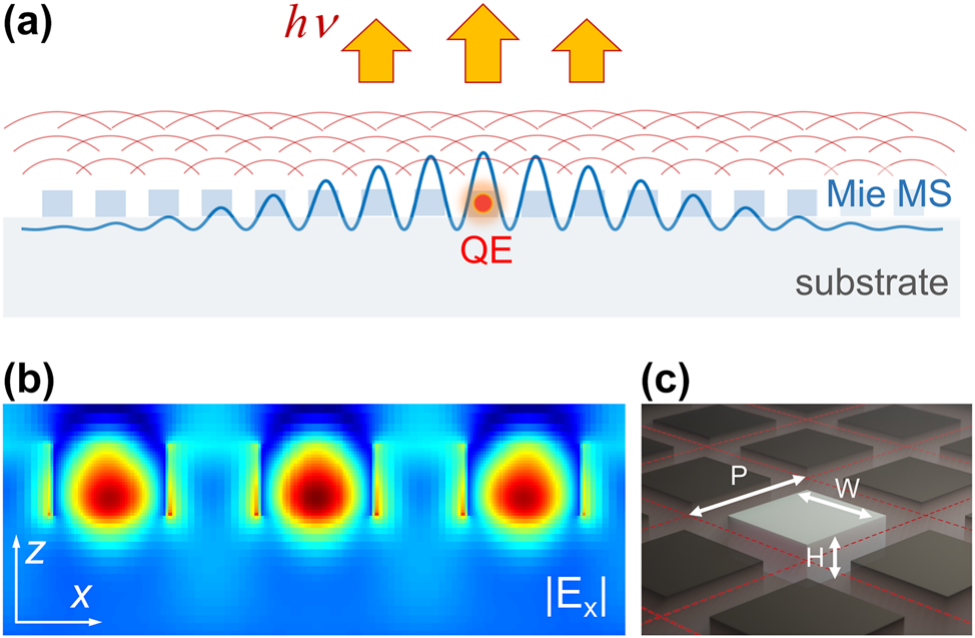
Overview of our Mie metasurface designs. (a) Illustration of Mie metasurface on a low-index dielectric substrate. A quantum emitter (QE) is embedded into one of the resonators. (b) Simulations of the in-plane electric field distribution within Al_0.4_Ga_0.6_As resonators at the ED mode wavelength (750 nm) excited by an *x*-polarised Gaussian beam. (c) Schematic illustration of the Mie metasurface consisting of rectangular dielectric resonators.

The fundamental modes in a dielectric resonator are Mie modes and the lowest order ones are the ED and MD modes. They weakly couple to free space, and therefore they can be excited by incident light, which creates regions of high electric field within the resonators and leads to enhanced light–matter interaction, for example, enhanced absorption [43]. Similarly, placing a QE at these regions can lead to more efficient coupling of single photon emission to free space (Figure 1a). The ED mode is characterised by a relatively uniform electric field within the resonator (Figure 1b), and therefore the ED mode can tolerate the randomness of the QE position. Furthermore, combining two Mie modes can also be used to control the directivity of emission, as it has been demonstrated in Huygens’ metasurfaces using the Kerker condition [19], [20], [21], [22], [23], [24], [25].

Behind the first metasurface design explored in this work is the Huygens’ principle with the metasurface in-plane ED and MD modes overlapping spectrally [34], [37]. To engineer the Huygens’ metasurface, we first determined how the ED and MD modes change their wavelengths with the metasurface parameters. The ED mode can be identified by its distinctive *E*-field distribution: it is confined within the resonator with the *E*-vector orientation primarily along one in-plane axis (Figure 1b) and the *H*-field rotating around it. Similarly, the MD has a *H*-field distribution with the *H*-vector orientation along an orthogonal in-plane axis (e.g. *H*_*y*_) and the *E*-field rotating around it [44]. These *E*_*x*_ and *H*_*y*_ components at the resonator centre therefore can be used to track the ED and MD mode wavelengths respectively.

**Figure 2:**
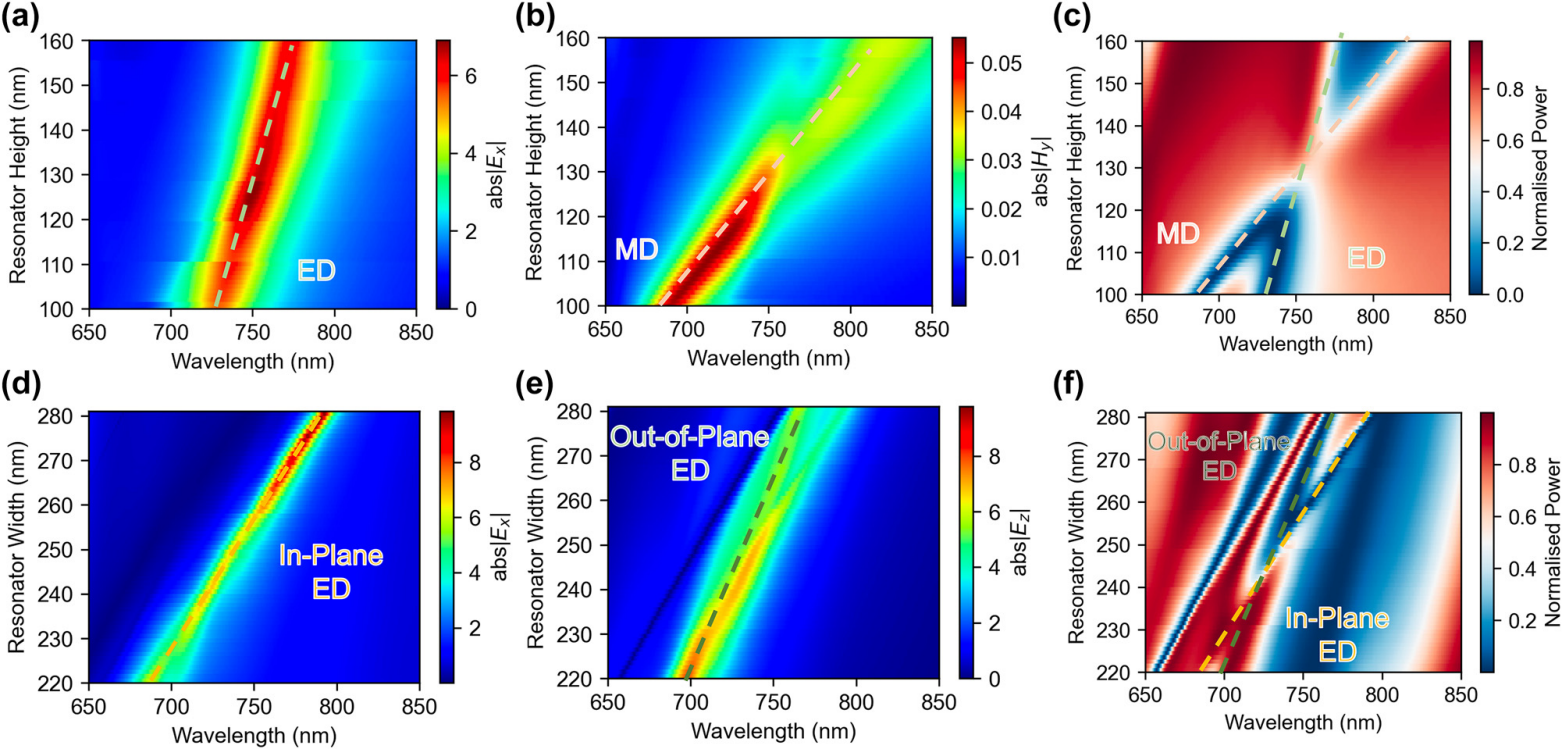
Tuning metasurface parameters to engineer the modal overlap at the quantum emitter (QE) emission wavelength. (a), (b) Tuning of the electric dipole (ED) mode wavelength (a) and the magnetic dipole mode (MD) wavelength (b) in the Huygens' metasurface made of Al_0.4_Ga_0.6_As resonators on a low index dielectric substrate (*n* = 1.45). The resonator height is used as a tuning parameter, the resonator period *p* = 450 nm and width *w* = 270 nm are kept constant. (c) Transmission map of the metasurface illustrating the overlap of the ED and MD modes at 750 nm for the resonator height of 135 nm. (d), (e) Tuning of the in-plane (d) and out-of-plane (e) ED mode wavelengths using the resonator width in the ED metasurface design. The *E*_*x*_ and *E*_*z*_ components spectra are simulated at the resonator centre for resonators of varying width when excited by a plane wave polarised along *x*-axis and incident at an angle of 10°. The in-plane and out-of-plane ED modes are marked with red and blue lines respectively. (f) Transmission map of the ED metasurface illustrating the overlap of the in-plane and out-of-plane ED modes for the resonator width of 257 nm.

We first simulated the metasurface as an infinite periodic array of Al_0.4_Ga_0.6_As resonators on a low index substrate (*n* = 1.45) using the finite difference time domain (FDTD) software (Lumerical) and periodic boundary conditions. We chose Al_0.4_Ga_0.6_As as the resonator material to make the simulations results directly applicable to semiconductor (e.g. GaAs) QDs monolithically embedded in an AlGaAs alloy. We found the condition of ED and MD mode overlap by varying the resonator dimensions (height *H*, width *W* and period *P*, Figure 1c). Among the metasurface parameters, the resonator height affects the wavelengths of the two modes differently, as illustrated in Figure 2a and b, and the tuning of this parameter allowed us to achieve an overlap between the ED and MD modes at 750 nm, a typical wavelength of QD emission [33].

To assist in selecting a metasurface with the overlapping ED and MD modes experimentally, we also modelled the metasurface transmission properties for varying resonator height. The wavelength of each mode can be identified by a drop in transmission (blue regions in Figure 2c), however when the ED and MD modes overlap spectrally, the transmission coefficient recovers to a higher value, as expected for a Huygens’ metasurface. The mode overlap therefore results in a distinctive spectral feature at a wavelength of 750 nm for the 135 nm tall metasurface (Figure 2c). We note that a small level of absorption is present in our design due to intrinsic absorption in the resonator [26].

The Huygens’ metasurface design primarily enhances outcoupling of photons polarised in the metasurface plane, as will be shown later. However, not all QEs exhibit such dipole moment orientation. Several types of QEs are known to generate photons with out-of-plane polarisation [38], [45], [46], and such emitters embedded in a uniform dielectric slab couple practically no photons into the air region due to total internal reflection within the slab. Therefore, for the QEs that primarily generate out-of-plane polarised photons, in the second metasurface design, which we refer to as the ED metasurface here, we adjusted the metasurface parameters to exploit the out-of-plane ED mode.

In this second design, the out-of-plane polarised photons can excite the out-of-plane ED mode and then couple to free space [44]. Furthermore, to enable the outcoupling of emission for both the in-plane and the out-of-plane polarisations, we aligned both the in-plane and out-of-plane ED modes to the wavelength of QE. To realise this design, we increased the height of the resonator to 180 nm and reduced the period and the width to 350 nm and 257 nm, respectively. To simulate the symmetric out-of-plane ED mode, we used a similar FDTD setup as for the Huygens’ metasurface, but with the incident light at a 10-degree angle (with Bloch boundary conditions). Figure 2d and e shows the tuning of the in-plane and out-of-plane ED modes with the resonator width: the two modes shift together as the resonator size changes, overlapping precisely at ∼750 nm for a resonator width of 257 nm (Figure 2f).

## 3 Results and discussion

**Figure 3:**
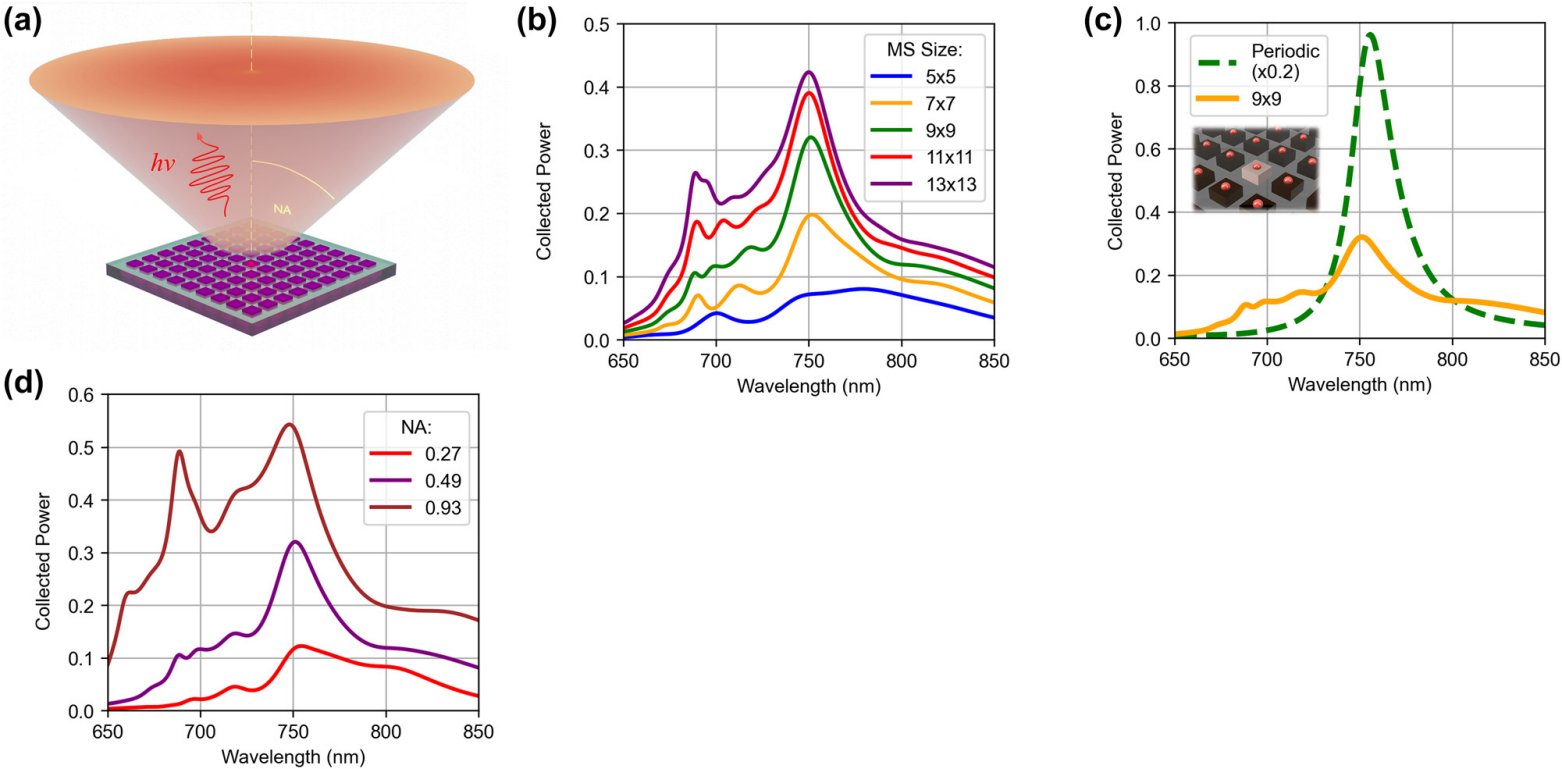
Efficiency of photon emission from an *x*-polarised dipole source in the centre of Huygens’ metasurface (MS) into the air region. Emission spectra are normalised to the total power emitted by the dipole in a uniform medium. (a) Illustration of the 9 × 9 Huygens’ MS with QE in the central resonator and the range of emission angles accounted in the efficiency calculations; (b) Effect of MS size (labelled by the number of elements) on emission properties; (c) Emission spectrum for the 9 × 9 Huygens’ MS with an embedded single QE compared to an emission spectrum for a MS with identical QEs in each Mie resonator (periodic simulations); (d) Effect of the NA of collection optics on the emission spectrum of the 9 × 9 Huygens’ MS (see illustration in a). The spectra in b–d include the effects of Huygens’ MS on Purcell enhancement and on directionality of emission.

To determine the effect of metasurface on an embedded QE we modelled the emitter by placing a point source with a broad spectrum of emission within a central resonator and simulated the energy flow away from the metasurface plane into the air region (Figure 3a). We also simulated the same point source placed in a uniform Al_0.4_Ga_0.6_As slab (of the same thickness as the metasurface) for comparison. The metasurface effect was then quantified by normalising the emission spectrum of the QE in the metasurface environment to the emission of the QE in the slab.

First, we determined a minimal size of the metasurface required to provide sufficiently accurate results in our simulations. We placed an *x*-polarised point source at the centre of the resonator in the middle of a 5 × 5 resonator array. A simulated emission spectrum into the air region is shown in Figure 3b. We then gradually increased the number of array elements to 13 × 13. We observed a relatively little difference in the emission spectra for the 11 × 11 and 13 × 13 cases, while the 9 × 9 resonator array was a suitable trade-off between accuracy and computing resources usage.

We note that simulations of the metasurface with periodic boundary conditions represents an unrealistic case where each resonator contains a QE. Simulations of the emission in this case are significantly different from the finite-area simulations results (Figure 3c). While both simulations showed an emission peak at 750 nm, the spectra of the finite arrays show a richer structure with additional features between 750 nm and 690 nm. More importantly, the intensity and the shape of the 750 nm peak were drastically different for the periodic simulations, confirming the need for the finite-area simulations for quantitative analysis.

Secondly, we defined a simulation geometry which represents typical experimental conditions for QE characterisation. Light from a QE tends to emit into a wide range of angles, whereas only photons emitted within the numerical aperture (NA) of a collecting lens are useful. In the simulations, we ‘collected’ only the light that passed through a 2D plane above the metasurface spanning the simulation region width (in the *xy*-plane). The distance of the plane from the metasurface, therefore, defines the collection angle and the effective NA. Since, a practical NA of the collection optics in experiments is ∼0.5 [33], we simulated emission spectra for this NA. A lower or higher NA changes the emission spectrum slightly, however the peak at ∼750 nm remains the dominant feature as illustrated in Figure 3d. We note that in Figure 3b the simulations of metasurfaces of different sizes are characterised by slightly different NA: NA = 0.49 for the 9 × 9 metasurface and NA = 0.56 for the 11 × 11 metasurface. Therefore, the power increase in Figure 3b is partially attributed to the increase in NA.

**Figure 4:**
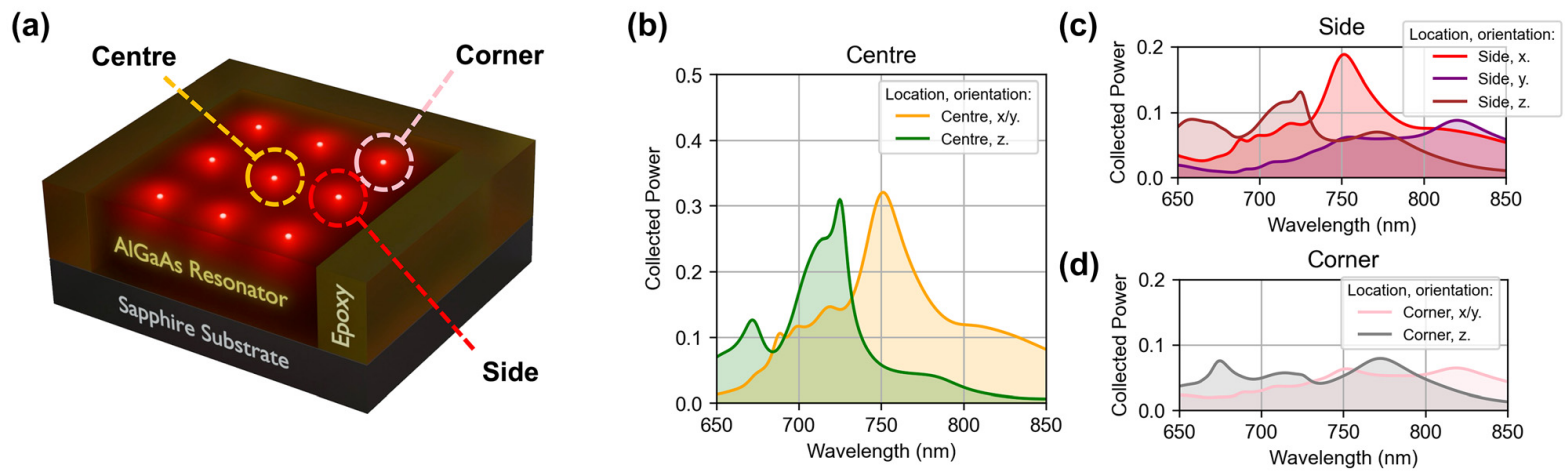
Effect of QE location and polarisation orientation on the efficiency of photon outcoupling from the 9 × 9 Huygens’ metasurface into the air region (NA = 0.5). Spectra are normalised to the total power emitted by a dipole in a uniform medium. a. Illustration of a central resonator showing nine QE locations with three unique locations highlighted (centre, side, corner). b–d. Emission spectra of the QE embedded in the centre (b), side (c) and corner (d) of the central resonator for *x/y* and *z* polarisations (normalised to a QE in a uniform medium).

Finally, we introduced the QE position and orientation randomness. The results in Figure 3 display the emission properties of the QE polarised along the *x*-axis and ideally positioned at the centre of the resonator. However, such a combination of polarisation and position is not representative of realistic systems, where the in-plane location of an emitter, e.g. a QD, and the polarisation of emitted photons are not pre-determined. To investigate and quantitatively evaluate the impact of QE position and polarisation, we simulated and averaged the emission for QEs at different locations in the resonator and with different point dipole polarisations, as shown in Figure 4. We assumed that the QE can be found in any of the nine locations, and that it can emit photons polarised along any directions with equal probabilities. While there are 27 possible combinations of the QE location and polarisation, only 7 are unique due to symmetry of the resonator: they are marked in Figure 4(a) as ‘Centre’ (*x-* and *z-*polarised), ‘Side’ (*x-*, *y-* and *z-*polarised) and ‘Corner’ (*x-* and *z-*polarised). We note that, if the QE is optically excited with above band gap, the pump intensity within the resonator could be non-uniform and it must be taken into account. However, for QDs at cryogenic temperatures, the photoexcited electrons can evenly redistribute within the resonator within a 1 ps time period for such small resonators, making intensity distribution effects on overall emission negligible.

The effect the QE position and polarisation on the emission spectrum is shown in Figure 4 for each of the 7 unique location/polarisation combinations. The spectra display a combined effect of directional emission and Purcell enhancement. A QE in the resonator centre with the polarisation axis in the *xy*-plane results in the most efficient emission into air with a peak at ∼750 nm (*orange line,* Figure 4b,): ∼33 % of the total number of generated photons are emitted into air within NA = 0.5. The peak at 750 nm matches the centre wavelength of the ED and MD modes. The ED mode, in particular, facilitates the outcoupling of emission due to the alignment of the ED mode amplitude maximum with the QE location. For a QE shifted towards the resonator side the efficiency drops to ∼18 % and 6 %, depending on the polarisation of the generated photon (Figure 4c, *red and purple lines*). This is consistent with a reduced ED mode amplitude at this location. For a QE shifted towards the resonator corner, the efficiency decreases further (∼6 %, averaged over the two polarisations) consistent with the mode amplitude in the corner (Figure 4d, *pink line*).

**Figure 5:**
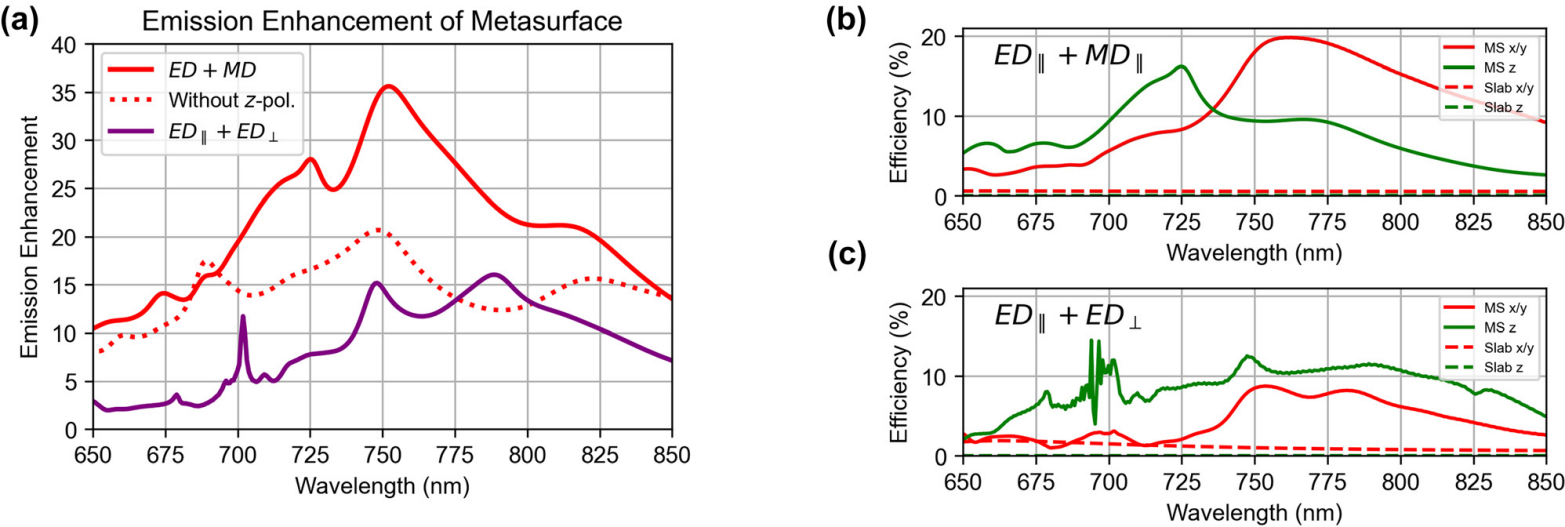
The order of magnitude emission enhancement provided by the two metasurface designs. a. Emission enhancement averaged over 27 QE location/polarisation combinations for the Huygens’ metasurface (MS) design (ED + MD) and for the metasurface with the in-plane/out-of-plane ED modes centred at 750 nm. The spectra are normalised to the averaged emission for the QE in uniform slabs of the same thickness as the MS. b, c. Average efficiency of photon outcoupling into the air regions (NA = 0.5) for the Huygens’ MS (b) and the ED MS (ED_||_ + ED_⊥_) (c).

Having found the emission spectra for each QE location/polarisation combination within the Huygens’ metasurface we can evaluate an average enhancement factor for all 27 cases compared to the average emission from a QE in the slab for the three principal polarisation orientations. The result shows that emission into free space from the QE embedded in the Huygens’ metasurface on average is enhanced by ∼35 times at the wavelength of the overlapping ED and MD modes (Figure 5a).

Next, we use the individual location/polarisation combinations to evaluate an enhancement factor for QEs with special emission properties, such as the recently reported local droplet etched (LDE) epitaxial GaAs QDs embedded in an AlGaAs Huygens’ metasurface [33]. These QDs are expected to emit photons primarily with the in-plane polarisation [38]. To account for their emission characteristics, we averaged the emission enhancement spectra for the in-plane polarised QEs only, excluding the 9 out-of-plane polarised cases. The result is shown as red dashed line in Figure 5a); the enhancement reaches ∼20 at the wavelength of the ED and MD modes (750 nm), in good agreement with the experimentally evaluated enhancement of above 10.

We note that the drop in average enhancement from ∼33 to ∼20 is not due to less efficient outcoupling for the in-plane polarised photons compared to the out-of-plane polarised photons. In fact, both the in-plane and out-of-plane polarised emission can outcouple from the Huygens’ metasurface into the air region with similar efficiencies, as shown in Figure 4b. The emission from the *z*-polarised QE embedded in the AlGaAs slab, however, produces almost no emission into free space (<0.1 %), because the *z*-polarised photons remain trapped in the slab due to the combination of the out-of-plane dipole emission pattern and total internal reflection within the slab. In contrast, in the metasurface, generated *z*-polarised photons can excite a metasurface mode, which then couples photons out into free space. As a result, the enhancement factor for only the *z*-polarised photons reaches a value over 100, whereas excluding the *z*-polarised emission from the average leads to a drop in the enhancement factor down to ∼20.

The proportions of *z*- and *x*-polarised generated photons, which emit from a QE placed at the resonator centre, up into the air region, are compared to the proportions trapped in the slab in Table 1. The results in the Table show that the Huygens’ metasurface facilitates the outcoupling of photons and reduces the loss of photons due to trapping in the slab from over 90 % percent down to several percent.

**Table 1:** Proportion of emission outcoupled into the air region and trapped in the slab and in the metasurface (MS) layer for both MS designs at the wavelength of 750 nm. The first (second) number shows the percentage emitted into NA = 0.9 (0.5). The Huygens’ MS (ED + MD) has a thickness of 135 nm, and the ED MS (ED_||_ + ED_⊥_) has a thickness of 180 nm.

Structure	Trapped emission (%)		Emission to air (%)	
	*x*/*y*	*z*	*x*/*y*	*z*
Slab (135 nm thick)	94	98	2 (1)	<0.1
MS1: ED + MD	6	5	35 (21)	25 (11)
Slab (180 nm thick)	87	>99	3 (1)	<0.1
MS2: ED_||_ + ED_⊥_	19	12	31 (12)	36 (21)

The Table also shows that at the Huygens’ point, the metasurface enables a smaller portion of *z*-polarised photons, 25 % for NA = 0.9, to outcouple into the air region, compared to the outcoupling efficiency of 35 % for the in-plane polarisation. For the practical NA of 0.5, these proportions reduce to 11 % and 21 % respectively. Although the 11 % efficiency is over two orders of magnitude higher compared to that for the slab, it can be increased in the second metasurface design by tuning the out-of-plane ED mode to the emission wavelength of 750 nm.

To quantify the effect of the spectrally overlapping in-plane and out-of-plane ED metasurface modes, we used the same analysis as for the Huygens’ metasurface: we placed a single dipole emitter in the central resonator of the 9 × 9 resonator array, and averaged and normalised the emission for the 9 locations and 3 polarisation orientations. The corresponding enhancement spectrum is shown as a purple curve in Figure 5a for the collection NA of ∼0.5. The maximum averaged enhancement for the ED metasurface design was ∼15. While the ED metasurface exhibited a smaller average enhancement in comparison to the Huygens’ metasurface, the outcoupling efficiency for the out-of-plane polarised emission relative to the in-plane polarised emission became greater throughout the considered spectral range (Figure 5b and c).

In the ideal case of QE in the resonator centre, the outcoupling efficiency reached 36 % for NA = 0.9 (Table 1), similar to the 35 % level for the in-plane polarised photons enabled by the Huygens’ metasurface. The ED metasurface design therefore may be of particular interest for QEs which emit out-of-plane polarised photons, such as epitaxial stacked QDs, quantum posts, and strained QDs [38], [39], [45], [46]. However, for QEs with random position and unknown polarisation properties, the Huygens’ metasurface provides on average a greater enhancement. We note that in this study QEs were positioned mid-height within the resonator, whereas adjusting the QE position along the *z*-axis can be used to further control the directionality of emission [34]. We also note that nanoscale fabrication of metasurfaces can deteriorate properties of QE resulting in, for example, emission linewidths broadening [47]. Nevertheless, nanofabricated metasurfaces with embedded QEs have been demonstrated to exhibit single-photon emission properties, such as an AlGaAs Huygens metasurface with LDE GaAs QDs [33].

Finally, we considered the Purcell factor for the QE in both metasurfaces to evaluate if the emission enhancement can be attributed to modification of the emission rate. The Purcell factor varied depending on the QE location/polarisation but averaged to approximately 85 % for both metasurfaces designs (see SI Figure 1), which is not significantly different than unity. Therefore, the number of photons emitted from the QE is approximately the same, whether the QE is in the metasurface or in a homogeneous environment (SI). The Mie metasurface effect can be interpreted as a modification of photon outcoupling without greatly impacting the emission rate of the QE: a generated photon can couple to a metasurface mode distributed over several resonators and then directionally couple to free space as illustrated in Figure 1a.

## 4 Conclusions

We numerically evaluated the effect of a Mie metasurface on emission from a single embedded QE and found that Mie resonances could be utilised to strongly enhance emission properties of isolated QEs. The Huygens’ metasurface design can provide over one order of magnitude enhancement in photon emission without the need for strict QE position alignment. The Purcell enhancement due to the Mie metasurface remains approximately unity, indicating that the metasurface provides efficient photon extraction without affecting the intrinsic QE emission rate. The ED modes are key for achieving the enhancement, offering a path for selective enhancement for photons emitted with a desired polarisation including the out-of-plane polarisation. Our simulation results reveal the fundamental underlying processes and modal coupling behind a recent experimental demonstration of emission enhancement from Huygens’ metasurfaces with single embedded QD emitters [33], allowing us to evaluate the role of the electric dipole modes and magnetic dipole modes within these metasurfaces and create a modelling process for a more general metasurface QE system. While the photonic design in this work represents an Al_0.4_Ga_0.6_As metasurface with integrated GaAs QDs, our findings can be extended to other material systems, such as InAs QDs formed in GaAs metasurfaces operating at the telecom wavelengths. We anticipate that the Mie metasurfaces will provide a scalable platform for single-photon sources, which requires lower tolerances in precision fabrication compared to alternate solutions, while offering polarisation-selective emission enhancement without a significant effect on the QE intrinsic emission rate.

## Supplementary Material

Supplementary Material Details
