# Supplementary material for: Mie metasurfaces for enhancing photon outcoupling from single embedded quantum emitters
Source: Nanophotonics. 2024 Oct 17;14(11):1917–25. doi: 10.1515/nanoph-2024-0300 (PMC12133310; doi:10.1515/nanoph-2024-0300)
Supplement: Supplementary file 1 — Supplementary Material Details [file j_nanoph-2024-0300_suppl_001.pdf]

Supplementary Information

Mie metasurfaces for enhancing photon outcoupling from single embedded quantum emitters

Samuel Prescott[1*], Prasad P Iyer[2,3], Sadhvikas Addamane[2,3], Hyunseung Jung[2,3], Ting S Luk[2,3], Igal Brener[2,3] and Oleg Mitrofanov[1,2*]

1. *University College London, Electronic and Electrical Engineering, London, WC1E 7JE, UK* 2. *Center for Integrated Nanotechnologies, Sandia National Lab, Albuquerque NM, USA,* 3. *Sandia National Laboratories, Albuquerque NM, USA.*

**samuel.prescott.21@ucl.ac.uk, o.mitrofanov@ucl.ac.uk*

Lumerical FDTD was used for all numerical electromagnetic simulations of metasurface (MS) properties. For characterising modes in MS design 1 (the Huygens' MS), periodic boundary conditions were used, with a broadband plane wave incident normally to the (MS). For MS design 2 (the spectrally overlapping in-plane and out-of-plane electric dipole modes), Bloch boundary conditions were used with a plane wave incident at 10 degrees to the MS normal. Both of these simulation setups collected transmission data using a transmission monitor plane placed below the MS.

The material system used in the simulation setups was $Al_xGa_{1-x}As$ resonators (where the Al proportion, x = 0.411) with n = 3.42 at a wavelength of 750 nm. The resonators were submerged in epoxy with n = 1.45 (so the epoxy was present on all sides of the resonator, except the resonator/air interface on the top surface of the MS), The resonator geometry was varied in simulation parameter sweeps to achieve the desired modal overlaps at an emission wavelength of approximately 750 nm, with the finalised parameters summarized in Table 1:

Design	MS Resonator Parameters (nm)		
	Resonator Pitch	Resonator Width	Resonator Height
Design **1** *Huygens' MS*	450	250	135
Design **2** *ED MS*	350	257	180

Table 1: MS parameters for the two MS designs, the resonator pitch, width and height in nm.

For emission characterisation and analysis, perfectly matched layer (PML) boundary conditions were used, with a simulation volume containing 9x9 (or a different quantity when specified) resonators, with a broadband single dipole source in the central resonator. As discussed in the paper, this source was moved for various quantum emitter (QE) positions and rotated for three QE dipole moment polarisations (x, y, and z polarised). The emission into the air was collected using a transmission monitor plane across the simulation region, above the MS. Equation 1 was used to approximate the collection angle (and therefore the effective

collection NA) using the distance of the transmission monitor above the top of the MS (d) and the width of the simulation region (w):

$$NA = sin(\theta), \text{ where } \theta = \tan^{-1}\left(\frac{w/2}{d}\right) \quad (1)$$

Fig. 1 shows the break-down of the Purcell factor for the various quantum emitter (QE) positions and polarisations in a central resonator of a finite-area FDTD simulation, for the two Mie MS designs discussed in the paper. The average in-plane Purcell enhancement for the ED + MD Huygens' MS design is 0.87, and for the $ED_{\parallel} + ED_{\perp}$ MS it is 0.86. The Purcell enhancement for a QE positioned precisely at the centre of the resonator, however, is approximately 1.5 for both designs.

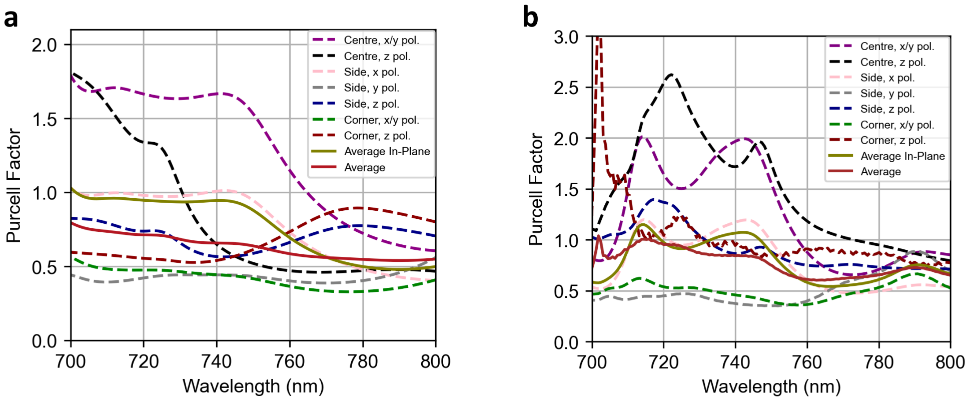

Figure 1: Purcell factor for the ED + MD Huygens' MS (**a**), and for the $ED_{\parallel} + ED_{\perp}$ MS (**b**), for emitters in various locations and polarisations.
